# Annotations.—Experiment in Vaccination

**Published:** 1893-07-29

**Authors:** 


### Annotations.

A Doctor, not a Doormat.

Doctors have been likened to buffers which prevent colliding railway carriages from smashing one another to pieces. They have also been described as doormats which always lie handy for every impatient or impudent fellow to wipe his feet upon. That is to say, the doctor is popularly supposed to be at anybody's beck and call, at any time of the day or night, and to be content with his fee whenever he can get it, or to be pleased whether he gets it or not. There is some truth in this supposition. The average doctor is really a good-natured, patient, forbearing man, who does all the good he can in the world, and takes cuffs and kicks—like a doctor! But sometimes the most patient of human beings will lift up a protesting hand, and a doctor at the Leeds County Court, the other day, set an example which other medical men may properly follow on suitable occasions. The doctor was the principal witness in the case; but before giving his evidence, he informed the judge that he had on some previous occasions given evidence in court, but when he had afterwards asked for his expenses he had been left to whistle for them. Whereupon the judge instructed him that he was entitled by law to claim his expenses before giving his evidence, and if the money were not forthcoming, he could decline to give evidence at all. The doctor did not need any prompting to take advantage of the law as laid down by the judge. He promptly left the court and the litigants in the lurch. Of course, there are cases in which such conduct would be both improper and wrong, but a very obvious rule may be laid down. Whenever a doctor is called upon to give evidence, he should consider whether the side which calls him is able to pay him; and if the litigant be able to pay, the doctor should insist upon being paid beforehand as the statute enacts. If the litigant be not able to pay, then the doctor must consider whether the case is important enough for him to give his time for nothing in the interests of public moralty and well being. In other words—If the litigant can pay, make him pay beforehand. If he cannot or will not pay, do notappear at all in his behalf unless the public interest, as well as the litigant's, demand a sacrifice. To this rule charity and common sense will suggest the fitting exceptions.

Thin Skins.

We are convinced that a too thin skin is a decided disadvantage; and yet the skins of all sorts of public and private persons seem to grow thinner year by year. The average doctor is so painfully sensitive that one might almost think he had no protecting cuticle at all but a mere *rete nervosa* exposed all bare and unprotected to a cruel world. A fortnight ago a person, whom nobody ever heard of, thought it desirable to write a letter to the *British Medical Journal* and to call in question the auditing of the accounts of the London and Counties Medical Protection Society. The writer of the letter was not a doctor, but a chartered accountant; and what he could have to do with the accounts of a private medical association beyond the mere desire to advertise himself as an accountant at the expense of the society no rational man could perceive. If ever there was a case for treating a person with absolute indifference and silence that surely was one. But no! Last week the two medical auditors and one of the secretaries of the society replied in the *Journal*, and filled nearly a column and a half in vindicating themselves against the entirely ridiculous criticisms of this absolutely unheard-of individual. That, in our judgment, was an exceedingly unwise and unbusinesslike course to pursue. It was encouraging impertinence. If a person has any sort of justifiable right to criticise, and if he criticise fairly and honourably, let his criticism be answered. But it is absurd to think of answering every pettifogger who loves the cheap notoriety of print. It is said that "a cat may look at a king." We freely admit it. But the converse is also a reasonable proposition, that the "King may decline to look at the cat." Doctors, as we have said before, live and work in the domestic circle. They need to mix more with men in order to counteract the effect of too exclusive an association with women and children. It is good for us all to rub shoulders with the common world. All sorts of persons take advantage of the thinness of the medical skin. The man or the society of honest purpose and established reputation can well afford to turn an indifferent back upon self-seeking and self-advertising critics.

A Striking Experiment in Vaccination.

At the close of last year (1892) a tramp from Edinburgh went to Ardlui in the West of Scotland, and there developed small-pox. He stayed for some days in a hut on the shores of Loch Long, a place well known to the present writer, and then left for Glasgow. In the hut with him were nineteen navvies and other persons, the navvies being there, along with a great many other navvies, for the purpose of constructing the new West Highland Railway. Now here was an interesting experiment: a hut full of navvies and others in a healthy district quite free from small-pox, is suddenly invaded by a person who remains there for several days among the nineteen, with small-pox developing upon him. What is the result? The result proved to be of the most striking and instructive character, at least, to persons who have open minds. Naturally the first question we ask about the nineteen persons in the hut is: Were they vaccinated? And how were they vaccinated? We give the answer in the words of Dr. McVail, D.P.H.Cantab., the Medical Officer of Health for the district: "Of the nineteen inmates of the hut at Ardlui three had already had small-pox, and none of these were attacked. Four had either been re-vaccinated or their primary vaccination had been performed subsequently to the age of twelve years, thus making it equivalent to re-vaccination, and none of these were attacked. Of the remaining twelve, one had four marks of vaccination, two had two marks, six had one mark, and two had no traces of ever having been vaccinated." Now, what happened to these twelve, well vaccinated, ill-vaccinated, or unvaccinated persons? "None of those who had more than one mark were attacked. Of the six who had one mark, two were attacked. Of the two who had no marks, both were attacked, and one died." That is to say, we have every stage, from sufficient vaccination to no vaccination at all, illustrated by experiment, and we find exactly what we should expect to find from our general knowledge of vaccination history. The three main points to be noted are that the well-vaccinated did not take small-pox at all; of the ill-vaccinated, one person in three took the disease, but did not die; of the non-vaccinated, all took it, and fifty per cent. died. This is a truly remarkable story; and if a sufficient number of examples of the kind could be intelligently put on record, the most sceptical anti-vaccinators would be compelled to intelligent conviction. Dr. McVail has done admirable work in this most important field of vaccination and small-pox, and we congratulate him upon his energy, intelligence, and perseverance.

